# Potential of Inducible Nitric Oxide Synthase as a Therapeutic Target for Allergen-Induced Airway Hyperresponsiveness: A Critical Connection to Nitric Oxide Levels and PARP Activity

**DOI:** 10.1155/2016/1984703

**Published:** 2016-07-20

**Authors:** Salome' V. Ibba, Mohamed A. Ghonim, Kusma Pyakurel, Matthew R. Lammi, Anil Mishra, A. Hamid Boulares

**Affiliations:** ^1^The Stanley Scott Cancer Center, School of Medicine, Louisiana State University Health Sciences Center, New Orleans, LA 70112, USA; ^2^Department of Biomedical and Clinical Sciences, University of Milan, Milan, Italy; ^3^Microbiology and Immunology Department, Faculty of Pharmacy, Al-Azhar University, Cairo, Egypt; ^4^Pulmonary/Critical Care and Allergy/Immunology Section, School of Medicine, Louisiana State University, New Orleans, LA 70112, USA; ^5^Eosinophilic Disorder Center in the Section of Pulmonary Diseases, Tulane University School of Medicine, New Orleans, LA 70112, USA

## Abstract

Although expression of inducible NO synthase (iNOS) in the lungs of asthmatics and associated nitrosative damage are established, iNOS failed as a therapeutic target for blocking airway hyperresponsiveness (AHR) and inflammation in asthmatics. This dichotomy calls for better strategies with which the enzyme is adequately targeted. Here, we confirm iNOS expression in the asthmatic lung with concomitant protein nitration and poly(ADP-ribose) polymerase (PARP) activation. We show, for the first time, that iNOS is highly expressed in peripheral blood mononuclear cells (PBMCs) of asthmatics with uncontrolled disease, which did not correspond to protein nitration. Selective iNOS inhibition with L-NIL protected against AHR upon acute, but not chronic, exposure to ovalbumin or house dust mite (HDM) in mice. Supplementation of NO by nitrite administration significantly blocked AHR in chronically HDM-exposed mice that were treated with L-NIL. Protection against chronic HDM exposure-induced AHR by olaparib-mediated PARP inhibition may be associated with the partial but not the complete blockade of iNOS expression. Indeed, L-NIL administration prevented olaparib-mediated protection against AHR in chronically HDM-exposed mice. Our study suggests that the amount of iNOS and NO are critical determinants in the modulation of AHR by selective iNOS inhibitors and renews the potential of iNOS as a therapeutic target for asthma.

## 1. Introduction

Asthma is a chronic disease characterized by airway inflammation and hyperresponsiveness (AHR), overproduction of mucus, and airway and vascular wall remodeling [1–4]. These manifestations lead to repeated periods of shortness of breath, wheezing, and chest tightness which may incapacitate affected individuals. The incidence of the disease is increasing at an alarming rate affecting 1 in 10 children and 1 in 12 adults with a total of 300 million worldwide [5]. Worldwide, deaths from asthma have reached over 250,000 annually. Asthma can be controlled by a combination of an inhaled corticosteroid (anti-inflammatory) and a short- or long-acting *β*2-adrenergic agonist. However, a sizable portion of these patients (~10%) do not respond to the available therapies [5, 6]. Thus, new therapies that target all or some of the symptoms of asthma are urgently needed.

An increasing number of conflicting reports have demonstrated detrimental, protective, and sometimes neutral roles for inducible NO synthase (iNOS) in the pathogenesis of asthma [7, 8]. However, it is undoubtedly established that iNOS is expressed in lungs of asthmatics with a subsequent production of NO and generation of the reactive metabolite ONOO^−^ [9–12]. It appears that expression of iNOS is even higher in sputum cells from asthmatics compared to those from patients with controlled disease or healthy individuals [13]. Thus, inhibition of iNOS appears to be a very viable therapeutic target to prevent manifestation of asthma symptoms upon exposure to allergens [14–16]. This potential has been challenged by the observation that a selective iNOS inhibitor did not affect airway inflammatory cell numbers or AHR after allergen challenge in steroid-naïve human asthmatics [17]. However, it is difficult to ignore the fact that asthma protection and susceptibility are associated with polymorphisms in the iNOS gene [18]. We recently showed that iNOS gene deletion was associated with a reduction in eosinophilia, mucus hypersecretion, and Th2 cytokine production upon an acute exposure to ovalbumin (OVA) [19]. Such protection was completely abolished upon a chronic exposure to the allergen. Interestingly, pulmonary fibrosis observed in wild type mice under the chronic protocol was completely absent in iNOS^−/−^ mice despite persistent IL-5 and IL-13 production. The published results exemplified the complexity of the role of iNOS in asthma and the preservation of its potential as a therapeutic target. We also showed that poly(ADP-ribose) polymerase- (PARP-) 1 is required for iNOS expression [20] and that PARP inhibition protects against AHR upon an acute or chronic exposure to allergens [21, 22]. Overall, we believe that it is premature to conclude that targeting iNOS in asthma is futile and that more studies should be geared toward exploring new avenues to take advantage of such an important clinical target. Accordingly, the goal of the present study was to examine whether pharmacological inhibition of iNOS could be manipulated to provide protection against AHR upon chronic OVA or house dust mite extracts (HDM) exposure and whether the protection conferred by PARP inhibition was related to its control of iNOS expression level.* L*-N6-(1-Iminoethyl)lysine dihydrochloride (L-NIL) and AZD2281 (olaparib), two clinically tested iNOS and PARP inhibitors, respectively, were used to conduct the following study.

## 2. Methods

### 2.1. Human Subjects, Immunohistochemistry, and Immunoblot Analysis

Five healthy and eight asthmatic individuals were recruited under a protocol (#8450) approved by institutional review board of Louisiana State University Health Sciences Center. Subjects were included in the study if they were ≥18 years of age with a physician diagnosis of asthma. Subjects were excluded if they were diagnosed with another lung disease other than asthma, had active malignancy or inflammatory condition, or smoked ≥10 pack-years. Asthma control was assessed by the Asthma Control Test (ACT) with a score ≤19 indicating uncontrolled asthma and ≥20 demonstrating that asthma is well controlled [23]. Human PBMCs isolated from peripheral blood of donors were subjected to protein extractions followed by immunoblot analysis with antibodies to nitrotyrosine (EMD Millipore, Bedford, MA), human iNOS (Abcam, Cambridge, MA), the poly(ADP-ribose) moiety (PAR) of PARP-modified proteins (ENZO Life Sciences, Farmingdale, NY), or GAPDH (Abcam). Paraffin-embedded tissue sections from two deidentified lung specimens from individuals who died from severe asthma were subjected to immunohistochemistry with antibodies to PAR, iNOS, or nitrotyrosine. The sections were then counterstained with hematoxylin and mounted prior to examination by light microscopy.

### 2.2. Animals, OVA and HDM Challenge, and AHR Measurements

Six-week-old to eight-week-old C57BL/6J male mice were purchased from Jackson Laboratories (Bar Harbor, ME, USA). C57BL/6 iNOS^−/−^ mice were bred at the LSUHSC vivarium and allowed unlimited access to sterilized chow and water. Husbandry, experimental protocols, and procedures were all approved by the LSUHSC Animal Care and Use Committee.

Mice were sensitized to chicken (3 mg/kg), OVA (Sigma-Aldrich, St. Louis, MO), or (0.5 *μ*g/kg) HDM (*Dermatophagoides pteronyssinus*) extract (Greer Labs, Lenoir, NC). The challenge protocols with OVA or HDM are outlined in Supplementary Figure S1 in Supplementary Martial available online at http://dx.doi.org/10.1155/2016/1984703. Briefly, mice were challenged with aerosolized 3% OVA for 30 min three times on days 14, 16, and 18 for the acute asthma model or three times a week for three weeks (chronic asthma model). Other groups of mice were challenged intranasally with 1.25 *μ*g/kg whole HDM extract on days 24, 25, and 26 for the acute asthma model or 3 times per week for a total of 4 weeks for the chronic asthma model. Control groups were not sensitized or challenged. Additional challenged groups of mice were administered* i.p.* 5 mg/kg* L*-N6-(1-Iminoethyl)lysine dihydrochloride (L-NIL) (Sigma-Aldrich) and/or olaparib (Selleckchem, Pittsburgh, PA) in saline 30 minutes after each challenge. Some groups of mice also received* i.p.* injections of 20 *μ*g/kg of nitrite (NaNO_2_) (Sigma-Aldrich) as NO source 30 min after each challenge.

AHR to inhaled methacholine was measured in unrestrained, conscious mice 24 h after the last challenge by recording “enhanced pause” (Penh) by whole body plethysmography (EMKA Systems, Falls Church, VA, or DSI Buxco, St. Paul, MN). In brief, the baseline readings were taken and averaged for 3 min after animals were placed in a barometric plethysmographic chamber. Normal saline or increasing concentrations of aerosolized methacholine (Sigma-Aldrich) (12.5–100 mg/mL) were nebulized, and readings were taken and averaged for 3 min after each nebulization and enhanced pause (*Penh*) representing AHR was calculated. Mice were sacrificed 24 h after the last challenge and lungs were subjected to RNA extraction as described below.

### 2.3. Cell Culture: Real Time PCR

Wild type or PARP-1^−/−^ mouse embryonic fibroblasts (MEFs) were serum starved for 24 hours and then treated with 1 *μ*g/mL LPS (Sigma-Aldrich) and 10 ng/mL IFN-*γ* (RD System Minneapolis, MN) for 6 hours. The collected cells as well as homogenized lungs from the abovementioned experimental groups were subjected to RNA extraction using the RNeasy mini kit (QIAGEN, Valencia, CA). The extracted RNA was reverse-transcribed and the resulting cDNA was subjected to quantitative real time PCR using primer sets (IDT, San Jose, CA, USA) specific to mouse* iNOS* (F 5′-GTG TTG CAA GCT GAT GGT CA-3′ and R 5′-TGT TGT AGC GCT GTG TGT CA-3′),* IL-5* (F 5′-GGGCTTCCTGCTCCTATCTA-3′ and R 5′- CAGTCATGGCA CAGTCTGAT-3′), or *β*-actin (F 5′-TAC AGC TTC ACC ACC ACA GC-3′ and R 5′-TCT CCA GGG AGG AAG AGG AT-3′).

### 2.4. Data Analysis

All data are expressed as means ± SD of values from at least 5 mice per group. PRISM software (GraphPad, San Diego, CA, USA) was used to analyze the differences between experimental groups by* t*-test or one way ANOVA followed by Tukey's multiple comparison tests.

## 3. Results 

### 3.1. PARP Activation, iNOS Expression, and Protein Nitration Are Elevated in Lung and PBMCs of Asthmatics

**Figure 1:**
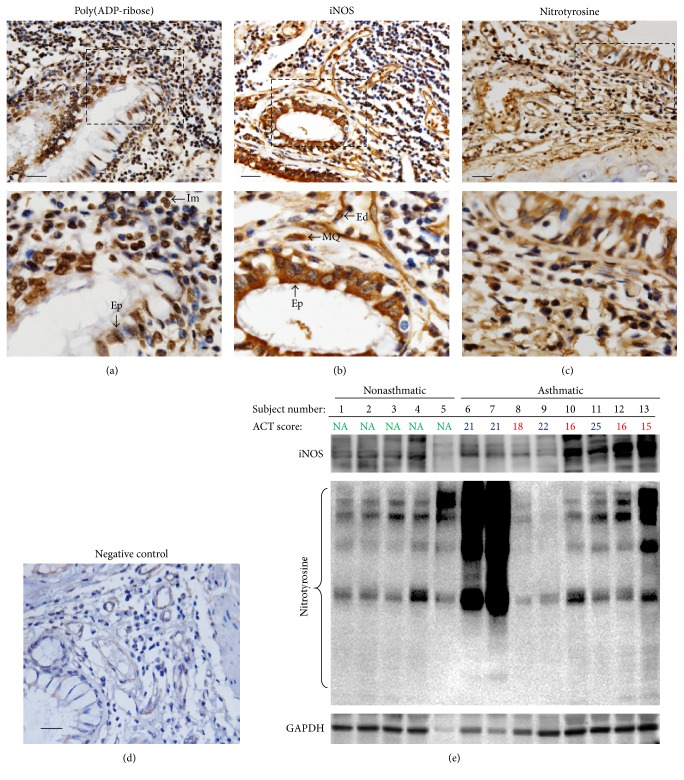
PARP activation, iNOS expression, and protein nitration in human asthmatics. Lung sections from a deidentified individual who died from severe asthma were subjected to immunohistochemistry with antibodies to PAR (a), iNOS (b), nitrotyrosine (c), or (d) control IgG followed by visualization with light microscopy. Bars: 20 *μ*m. The lower panels represent magnifications of the boxed areas. Ed, endothelial cells; Ep, epithelial cells; Im, immune cells; MQ, macrophages. (e) PBMCs collected from asthmatics with controlled (ACT score ≥ 20) or uncontrolled (ACT score ≤ 19) disease or healthy nonasthmatic (NA) volunteers were subjected to protein extraction followed by immunoblot analysis with antibodies to human iNOS, nitrotyrosine, or GAPDH.

We recently showed that PARP is activated in lung tissues and PBMCs of human asthmatics [21].  Figure 1(a) confirms the activation of PARP in lung tissues by immunohistochemistry using antibodies to the poly(ADP-ribose) moiety of modified proteins. The figure also shows that PARP activation occurred in epithelial and a subpopulation of immune cells.  Figure 1(b) shows that iNOS expression is prominent in epithelial and endothelial cells and macrophages. Protein nitration as assessed by IHC with antibodies to nitrotyrosine appears to be distributed throughout the lung tissue including the matrix but was more prominent in epithelial cells (Figure 1(c)).

PBMCs collected from asthmatics or healthy individuals were subjected to immunoblot analysis with antibodies to nitrotyrosine, iNOS, or GAPDH.  Figure 1(d) shows that iNOS is highly expressed in PBMCs from asthmatics compared to cells from healthy individuals. However, the expression of iNOS did not strictly correspond to protein nitration. Indeed, some PBMCs exhibited high levels of iNOS but showed protein nitration levels comparable to those detected in cells from nonasthmatics. Conversely, PBMCs that exhibited extensive protein nitration displayed low levels of iNOS. Interestingly, the two samples (6 and 7) that displayed high levels of protein nitration were collected from patients whose asthma was under control according to their ACT score of 21. However, samples from uncontrolled asthma (10 and 12 with ACT scores = 16) displayed levels of protein nitration comparable to those from nonasthmatics but with high levels of iNOS. Overall, these results exemplify the known complexity of the relationship between iNOS, protein nitration, and asthma in humans. Nevertheless, it is noteworthy that the presence of these factors does not necessarily mean a critical function in the disease but results from our laboratory and many others have suggested a potentially direct role for PARP and iNOS in some or most aspects of asthma.

### 3.2. Differential Protection of iNOS Inhibition against AHR Manifestation upon Acute and Chronic Exposure to OVA in Mice

**Figure 2:**
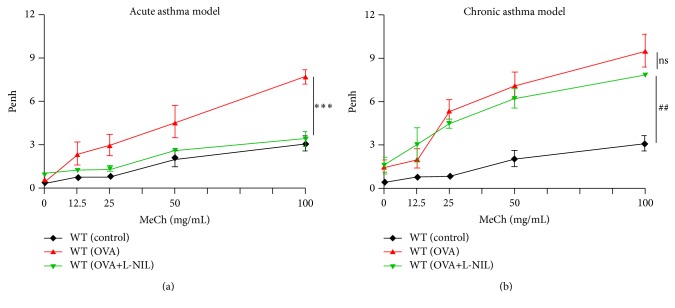
Effect of iNOS inhibition by L-NIL on AHR manifestation upon acute or chronic exposure to OVA in mice. C57BL/6 mice were subjected to OVA sensitization followed by an acute (a) or chronic (b) challenge to aerosolized OVA or left unchallenged. A group of WT mice were administered i.p. 5 mg/kg of the specific iNOS inhibitor (L-NIL) or saline 30 min after each OVA challenge.* Penh* was recorded 24 h after the last challenge using a whole body plethysmograph system before and after the indicated concentrations of aerosolized methacholine (MeCh). Results are plotted as maximal fold increase of* Penh* relative to baseline (0 *μ*M MeCh) and expressed as mean ± SEM, where *n* = 5 mice per group. *∗∗∗*, difference from OVA-challenged mice; *p* ≤ 0.001. ##, difference from control unchallenged mice; *p* ≤ 0.01; ns, no significant difference.

Our laboratory has shown that iNOS gene deletion is protective against airway inflammation upon acute, but not chronic, exposures to OVA [19]. Interestingly, such gene deletion prevented lung fibrosis in the chronic model of the disease. Given the potential connection between, and the coexistence of, lung fibrosis and AHR in chronic asthma [24], we explored the possibility that administration of L-NIL, a clinically tested iNOS inhibitor, may be protective against AHR upon both acute and chronic exposures to OVA in mice. L-NIL is a selective and long acting inhibitor of iNOS with IC_50_ = 3.3 *μ*M for mouse iNOS [25]. A clinical trial conducted by Barnes group [14] showed that administration of 200 mg of L-NIL reduced exhaled NO in patients with mild-to-moderate asthma to levels lower than those detected in placebo-administered healthy subjects as early as 30 min after administration. Mice were subjected to the acute or chronic model of asthma as described in Supplementary Figure S1 followed by an assessment of AHR using full body plethysmography.  Figure 2(a) shows that L-NIL administration at a dose of 5 mg/kg was very effective in blocking the manifestation of AHR upon acute exposure to OVA. Surprisingly, however, the protection achieved by L-NIL administration was completely lost when mice were chronically exposed to OVA (Figure 2(b)). Similar differential results were achieved using iNOS^−/−^ mice that were sensitized and acutely (Supplementary Figure S2A) or chronically (Supplementary Figure S2B) challenged to OVA. The effects of iNOS inhibition on AHR were similar to the differential protection conferred by iNOS gene deletion against acute versus chronic airway inflammation reported by us [19].

### 3.3. Inhibition of iNOS by L-NIL Failed to Protect against AHR Induced by a Chronic Exposure to HDM, Which Is Reversed upon NO Supplementation by Nitrite Administration

**Figure 3:**
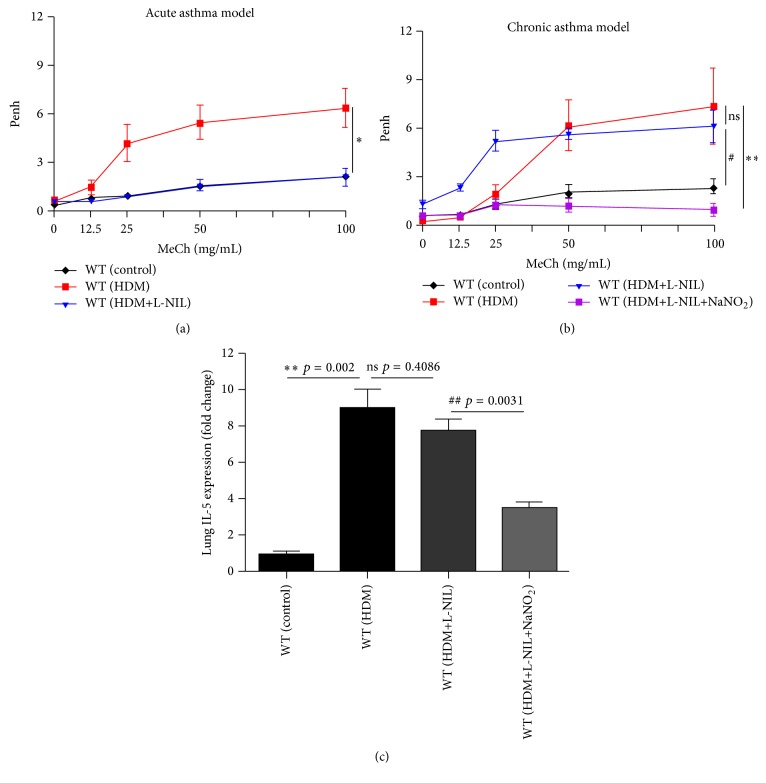
Effect of iNOS inhibition by L-NIL on AHR manifestation upon exposure to HDM in mice and the influence of NO supplementation by nitrite administration. C57BL/6 mice were subjected to HDM sensitization followed by acute (a) or chronic (b) intranasal challenge with HDM or were left unchallenged. Challenged mice were administered* i.p.* 5 mg/kg L-NIL with or without 20 mg/kg of nitrite (NaNO_2_) as NO source 30 min after each HDM challenge.* Penh* was recorded 24 h after the last HDM challenge using a whole body plethysmograph before and after the indicated concentrations of aerosolized MeCh. Results are plotted as maximal fold increase of* Penh* relative to baseline and expressed as mean ± SEM, where *n* = 5 mice per group. *∗* and *∗∗*, difference from HDM challenged mice with *p* ≤ 0.05 and *P* ≤ 0.01, respectively; #, difference from control unchallenged mice with *p* < 0.05. RNA isolated from the lungs of the different experimental groups was reverse-transcribed and the resulting cDNA was subjected to real-time PCR with primer sets specific to mouse IL-5 or *β*-actin. Data is expressed as fold change of values detected in lungs of control mice. *∗∗*, difference from control unchallenged mice with *p* = 0.002; ##, difference from HDM-exposed mice receiving L-NIL with *p* = 0.0031. ns, nonsignificant difference with *p* = 0.4086.

Given the clinical relevance of the present studies and the limitation of the OVA models, we elected to use HDM to induce asthma in mice due to its characteristic as a major allergen for humans [21]. To this end, mice were sensitized to HDM and then subjected to intranasal exposures to the allergen either acutely constituted by simultaneous daily exposures for 3 days or chronically by challenging the animals three times a week for four weeks as described in Supplementary Figure S1.  Figure 3(a) shows that, similar to the acute OVA model, L-NIL administration was extremely efficient in blocking HDM-induced AHR; in fact, AHR of HDM-treated mice that received the drug was identical to animals that were not exposed to HDM. Contrary to the acute HDM exposure model, iNOS inhibition by L-NIL did not provide a significant protection against AHR upon a chronic exposure to HDM. Altogether, the differential effects of iNOS inhibition on AHR induced by acute or chronic HDM exposure were very similar to those observed using the acute and chronic OVA models of allergic lung inflammation. These results also demonstrate that the role of iNOS in AHR manifestation is not specific to a given model and may be considered as a general phenomenon.

We next examined whether the failure of iNOS inhibition by L-NIL administration can be reversed by mere administration of NO source such as nitrite.  Figure 3(b) shows that administration of 20 *μ*g/kg nitrite,* i.p.*, 30 min after each challenge with HDM exerted a remarkable protection against AHR manifestation in response to increasing doses of methacholine. Concomitantly, nitrite supplementation decreased Th2 inflammation as assessed by IL-5 mRNA in the lung (Figure 3(c)). Given the fact that nitrite administration provides moderate levels of NO [26], these results suggest that NO levels are an important determinant for the protection against AHR and Th2 inflammation.

### 3.4. PARP-1 Inhibition-Mediated Protection against AHR in Chronically HDM-Exposed Mice is Lost upon L-NIL Administration

**Figure 4:**
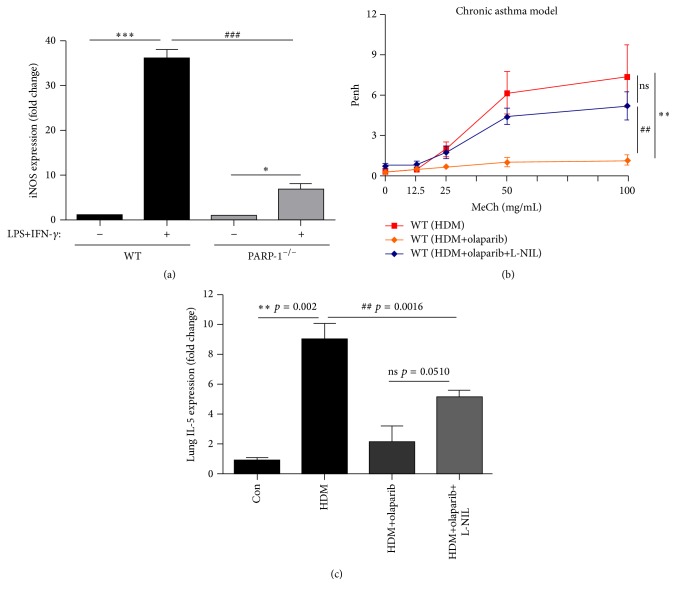
Effects of PARP inhibition on iNOS expression in LPS/IFN-*γ*-treated MEFs and of iNOS inhibition on the protection conferred by the PARP inhibitor olaparib against AHR in chronically HDM-exposed mice. (a) MEFs derived from WT or PARP-1^−/−^ mice were treated with a combination of 1 *μ*g/mL LPS and 10 ng/mL IFN-*γ*. RNA was isolated after 6 hours of treatment and reverse-transcribed to generate cDNA, which was subjected to quantitative PCR with primers specific to mouse iNOS or *β*-actin. The data is expressed as fold change normalized to levels of *β*-actin. *∗* and *∗∗∗*, difference from nonstimulated cells with *p* ≤ 0.05 and *p* ≤ 0.001, respectively; ###, difference from LPS/IFN-*γ*-treated WT cells with *p* ≤ 0.001. (b) WT mice were subjected to HDM sensitization followed by a chronic i.n. challenge with HDM or left unchallenged. Challenged mice were administered i.p. 5 mg/kg olaparib with or without 5 mg/kg of L-NIL 30 min after each HDM challenge.* Penh* was recorded 24 h after the last challenge in response to increasing doses of MeCh. Results are plotted as maximal fold increase of* Penh* relative to baseline and expressed as mean ± SEM, where *n* = 5 mice per group. *∗∗*, difference from HDM challenged mice; ##, difference from the group that received 5 mg/kg olaparib; *p* ≤ 0.01. RNA isolated from the lungs of the different experimental groups was reverse-transcribed and the resulting cDNA was subjected to real time PCR with primer sets specific to mouse IL-5 or *β*-actin. Data is expressed as fold change of values detected in lungs of control mice. *∗∗*, difference from control unchallenged mice; ##, difference from HDM-exposed mice receiving olaparib; ns, no significant difference. Note that values of control and HDM-exposed mice are the same as those described for Figure 3(c).

We previously demonstrated that PARP inhibition, genetically or by olaparib, protects against asthma manifestation including airway inflammation and AHR [21, 22]. We also showed that PARP-1 regulates iNOS expression in an animal model of asthma and in cultured cells [20]. The most fascinating aspect of the connection between PARP-1 and iNOS is that PARP-1 inhibition does not completely inhibit expression of iNOS [20] as demonstrated in results shown in Figure 4(a) using lung fibroblasts isolated from WT and PARP-1^−/−^ mice and treated with a combination of LPS and IFN-*γ*. Such incomplete inhibition was also observed in endothelial cells (data not shown). We thus hypothesized that the beneficial effect of PARP-1 inhibition on AHR may be associated with the partial reduction in iNOS expression, which presumably leads to a production of low-to-moderate levels of NO. Accordingly, inhibition of iNOS by L-NIL treatment was predicted to abrogate the protective effect of PARP inhibition. To test this hypothesis, mice were chronically exposed to HDM and were administered a combination of 5 mg/kg olaparib and 5 mg/kg L-NIL after each challenge.  Figure 4(b) shows that olaparib treatment provided an excellent protection against AHR, which was significantly abolished by L-NIL administration. Unlike its effect on AHR, L-NIL administration only partially reversed olaparib-mediated protection against Th2 inflammation as assessed by IL-5 mRNA in lungs (Figure 4(c)). These results suggest that the low expression of iNOS observed upon PARP inhibition was protective against both AHR and Th2 inflammation.

## 4. Discussion

The viability of iNOS as a therapeutic target for the treatment of asthma was hindered by the contradictory reports of a number of encouraging animal studies and the negative results of a clinical trial. The results of the trial showed that a selective iNOS inhibitor similar to the one used in the current study provided no protection against AHR or airway inflammation after allergen challenge in steroid-naïve human asthmatics [14]. A detailed study from our laboratory showed that iNOS exerted a differential effect on acute and chronic asthma [19]. iNOS inhibition was effective in blocking only acute allergic inflammation but failed to provide any protection against chronic inflammation [19]. The effects on chronic inflammation were consistent with those reported by Singh et al. using a selective iNOS inhibitor (GW274150) [17]. Interestingly, however, iNOS inhibition protected against airway remodeling potentially by preventing an increase in TIMP-2 expression [19] despite the persistence of inflammation. These results suggest that iNOS inhibition may be protective against some aspects of asthma but not others. The present study represents a continuation of our effort to understand the role of iNOS in asthma and explore new mechanisms by which the enzyme can be targeted for therapy against the disease. What is certain is that the enzyme plays important roles in the disease; what is lacking, however, is the strategy by which it can be adequately targeted to achieve an efficacious clinical outcome. We believe that the results of the present study renew the potential of iNOS as a therapeutic target for asthma with a specific consideration to the levels of NO in patients treated with selective iNOS inhibitors.

High levels of iNOS and ONOO^−^, the reactive byproduct of NO, are undoubtedly deleterious and participate in the pathology of asthma [19, 27, 28] as well as many chronic inflammatory diseases [9–12]. Sugiura et al. showed that patients with refractory asthma have even higher levels of iNOS and protein nitration in cells collected from their sputum than those from patients with well-controlled asthma [29]. This connection was recently strengthened by a study demonstrating that an iNOS-Dual oxidase-2-thyroid peroxidase metabolome is the basis of nitrogen radicals and subsequent protein nitration in human severe asthma [30]. Our results show that the elevated iNOS expression is also observed in PBMCs of asthmatics and potentially even higher in cells from asthmatics with uncontrolled disease. Surprisingly, this is the first report examining the levels of iNOS and protein nitration in PBMCs of asthmatics. Our results suggest that the role of iNOS in the pathogenesis of the disease may stem from circulating cells in addition to those in the lung. Although the cohort size is small in the present study, our results show a potential disconnect between expression of iNOS and protein nitration within the same cells suggesting that PBMCs may not be the major target of NO and its byproducts. This disconnect does not appear to exist in cells derived from either bronchial lavage fluids or sputum as shown by Yamamoto et al. and Sugiura et al., respectively [13, 29]. Altogether, the results of these studies and many others including ours [19, 20] predict that iNOS may be an ideal target for the treatment of asthma. Surprisingly, targeting this enzyme has been unsuccessful [17]. We speculated that our understanding of the role of NO and iNOS in asthma may not be sufficient to allow a better approach to achieve the desirable clinical outcomes using selective iNOS inhibitors.

In the current study, we show that iNOS inhibition, by the selective inhibitor L-NIL and confirmed by the use of a gene knockout approach, provided an excellent protection against AHR upon an acute exposure but not upon a chronic exposure to allergens in two experimental asthma models. These results are rather similar to the differential protection provided by iNOS inhibition against airway inflammation. It is noteworthy that NO supplementation aggravated Th2 inflammation in HDM-exposed mice as assessed by lung IL-5 mRNA levels. At this stage it is not clear whether the protection and loss of protection in the acute and chronic models, respectively, are associated with the status of inflammation as manifestation of AHR is not always strictly related to inflammation in human asthmatics [31]. The interesting aspect of this study is that the protection was recovered in the chronic HDM asthma model after NO supplementation by nitrite administration. The dose of nitrite used in this study was shown to deliver low-to-moderate levels of NO and protects against several oxidative stress-related conditions including hypoxic vasodilation, ischemia of the heart and liver, and postoperative ileus [26, 32, 33]. It is important to acknowledge that the failure of iNOS inhibition to protect against AHR in chronic asthma may be related to other factors besides NO; however, the level of the gas may constitute a major determinant in the protection against AHR. It is also important to acknowledge that one of the limitations of the current study is that the levels of NO upon nitrate supplementation were not measured. We predict that these levels might not have reached those in HDM-exposed mice; otherwise, we would expect a failure of protection against AHR manifestation. An additional limitation is the quantification of Penh using whole body plethysmography to measure AHR. Although measuring lung resistance is regarded as a better means to assess lung function, our previous studies demonstrate, for instance, a similar protection against AHR by PARP inhibitors using either method [22, 34]. Additionally, Penh has been demonstrated to correlate well with invasive measures of AHR [35].

Our laboratory established a reciprocal relationship between iNOS and PARP-1 [20]. PARP-1 activity and expression are required for iNOS expression [36]. Such regulation is linked to the dependence of iNOS gene on NF-*κ*B and control of the activity as well as the subcellular trafficking of the transcription factor by PARP-1. Interestingly, PARP inhibition, pharmacologically or by gene knockout, protected against inflammation and AHR both upon acute and chronic exposures to either OVA or HDM. Such protection occurred despite the fact that iNOS expression is markedly reduced upon PARP inhibition. It is interesting, however, that PARP-1 inhibition does not completely abrogate expression of iNOS. This partial inhibition may allow for only low-to-moderate level production of NO and constitutes the basis for the persistent protection against AHR conferred by PARP inhibition even after a chronic exposure to allergens.

## 5. Conclusions

Our results provide additional support for the hypothesis that the amount of iNOS and NO are critical determinants in the modulation of AHR by selective iNOS inhibitors and may also help explain why the clinical study by Singh et al. [17] found that GW274150 failed to protect against AHR. It is noteworthy that the levels of exhaled NO achieved upon administration of the inhibitor reached amounts lower than those observed in healthy nonsmoker and nonatopic subjects as comprehensively reviewed by Dweik et al. [37]. The severe reduction in the levels of NO in the study by Singh et al. may be detrimental and as such prevention of AHR might not have been possible. Furthermore, the drug was delivered orally, which may be another limitation of the study. These speculations and the positive results of our study on the role of iNOS and the critical influence of NO levels on AHR should lead to reevaluation of the benefit of selective iNOS inhibitors in blocking AHR in human asthmatics, especially those with uncontrolled disease.

## Supplementary Material

Figure S1: mice were challenged with aerosolized 3% OVA for 30 min three times on days 14, 16, and 18 for the acute asthma model or three times a week for three weeks (chronic asthma model). Other groups of mice were challenged intranasally with 1.25 μg/kg whole HDM extract on days 24, 25, and 26 for the acute asthma model or 3 times per week for a total of 4 weeks for the chronic asthma model. Control groups were not sensitized or challenged. Additional challenged groups of mice were administered *i.p.* 5 mg/kg *L*-N6-(1-Iminoethyl)lysine dihydrochloride (L-NIL) (Sigma-Aldrich) and/or olaparib (Selleckchem, Pittsburgh, PA) in saline 30 minutes after each challenge. Some groups of mice also received *i.p.* injections of 20 μg/kg of nitrite (NaNO_2_) (Sigma-Aldrich) as NO source 30 min after each challenge.
Figure S2: WT or iNOS^−/−^ mice were subjected to OVA sensitization followed by the acute (A) or chronic (B) OVA challenge protocol as described for Figure 2. Penh was recorded 24 h after the last challenge in response to increasing doses of aerosolized MeCh. Results are plotted as maximal fold increase of Penh relative to baseline (0 mM MeCh) and expressed as mean ± SEM where n=5 mice per group. ∗, difference from HDM challenged mice; #, difference from control unchallenged mice p < 0.05. The data attained using L-NIL is included for comparison.

## Abbreviations

AHR: Airway hyperresponsiveness
HDM: House dust mite extract
iNOS: Inducible NO synthase
L-NIL: * L*-N6-(1-Iminoethyl)lysine dihydrochloride
PARP-1: Poly(ADP-ribose) polymerase-1
PBMCs: Peripheral blood mononuclear cells
Penh: Enhanced pause.

## Additional Points

The results of the present study suggest that the amount of nitric oxide may be critical for the protection against airway hyperresponsiveness and renew the potential of iNOS as a therapeutic target for the treatment of asthma.
