# Supplementary material for: Potential of Inducible Nitric Oxide Synthase as a Therapeutic Target for Allergen-Induced Airway Hyperresponsiveness: A Critical Connection to Nitric Oxide Levels and PARP Activity
Source: Mediators Inflamm. 2016 Jul 20;2016:1984703. doi: 10.1155/2016/1984703 (PMC4971330; doi:10.1155/2016/1984703)
Supplement: Supplementary file 1 — Figure S1: mice were challenged with aerosolized 3% OVA for 30 min three times on days 14, 16, and 18 for the acute asthma model or three times a week for three weeks (chronic asthma model). Other groups of mice were challenged intranasally with 1.25 μg/kg whole HDM extract on days 24, 25, and 26 for the acute asthma model or 3 times per week for a total of 4 weeks for the chronic asthma model. Control groups were not sensitized or challenged. Additional challenged groups of mice were administered i.p. 5 mg/kg L-N6-(1-Iminoethyl)lysine dihydrochloride (L-NIL) (Sigma-Aldrich) and/or olaparib (Selleckchem, Pittsburgh, PA) in saline 30 minutes after each challenge. Some groups of mice also received i.p. injections of 20 μg/kg of nitrite (NaNO2) (Sigma-Aldrich) as NO source 30 min after each challenge. Figure S2: WT or iNOS−/− mice were subjected to OVA sensitization followed by the acute (A) or chronic (B) OVA challenge protocol as described for Figure 2. Penh was recorded 24 h after the last challenge in response to increasing doses of aerosolized MeCh. Results are plotted as maximal fold increase of Penh relative to baseline (0 mM MeCh) and expressed as mean ± SEM where n=5 mice per group. ∗, difference from HDM challenged mice; #, difference from control unchallenged mice p < 0.05. The data attained using L-NIL is included for comparison. [file 1984703.f1.pdf]

Supplementary Figure S1

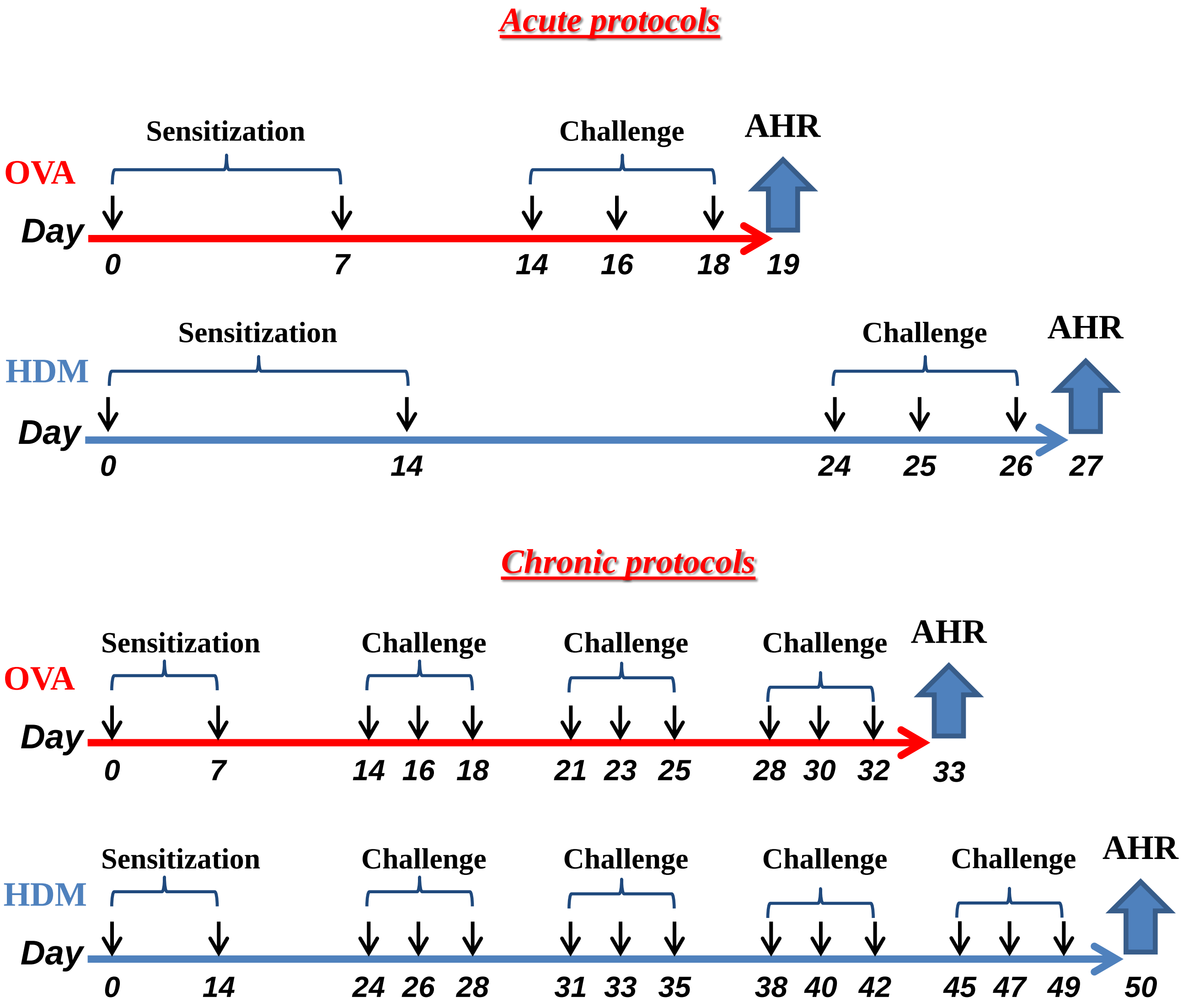

Supplementary Figure S2

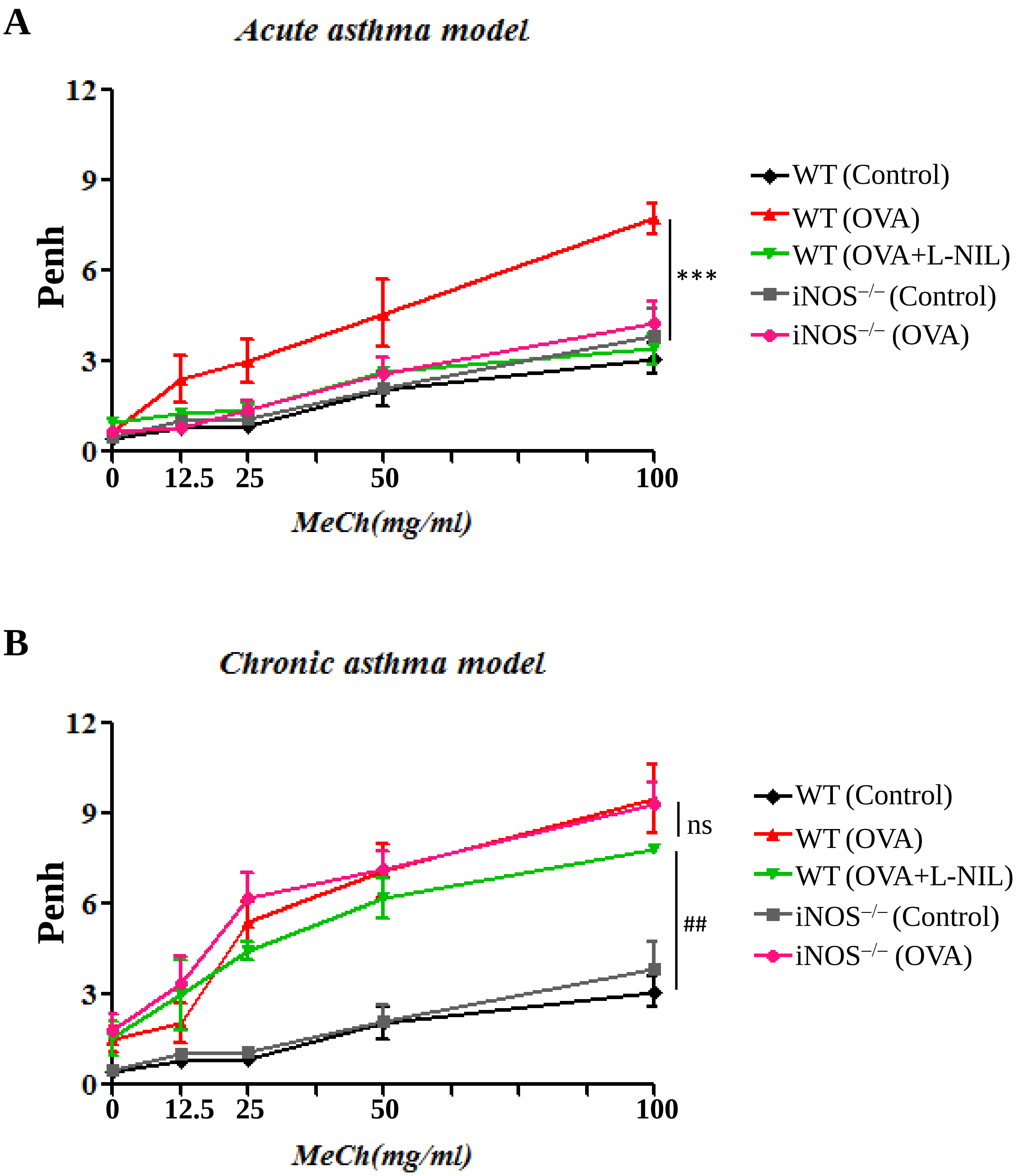

WT or iNOS$^{-/-}$ mice were subjected to OVA sensitization followed by the acute (A) or chronic (B) OVA challenge protocol as described for Figure 2. *Penh* was recorded 24 h after the last challenge in response to increasing doses of aerosolized MeCh. Results are plotted as maximal fold increase of *Penh* relative to baseline (0 mM MeCh) and expressed as mean ± SEM where n=5 mice per group. *, difference from HDM challenged mice; #, difference from control unchallenged mice $p < 0.05$. The data attained using L-NIL is included for comparison.
